# Past and Future of Vaccinations: From Jenner to Nanovaccinology

**DOI:** 10.3390/vaccines11020384

**Published:** 2023-02-07

**Authors:** Alessio Facciolà, Giuseppa Visalli

**Affiliations:** Department of Biomedical and Dental Sciences and Morphofunctional Imaging, University of Messina, 98125 Messina, Italy

Undoubtedly, vaccines are the most effective and safe weapons available to public health for the primary prevention of infectious diseases. The introduction of vaccinations has made it possible over time to reduce the spread of some serious and fatal diseases or even to eliminate them in certain countries, radically changing the epidemiological scenario of vaccine-preventable infectious diseases (VPDs), which represented for centuries the greatest scourge of humanity, and the whole human history. In just two centuries, there has been the achievement of exceptional results never obtained with other therapeutic and/or preventive treatments, such as the global eradication of smallpox and, in some countries, of some other diseases such as poliomyelitis and diphtheria [1,2]. Moreover, a drastic reduction in the frequency of these pathologies and, at the same time, in complications, hospital admissions, and deaths has occurred in developed countries [3]. Even if the general improvement in hygienic-sanitary conditions has greatly contributed to this success, it is essential to emphasize the fundamental role played by vaccinations in achieving these incredible goals, thanks also to the community protection that they determine (“herd immunity”), a public and community good from which everyone has the right to benefit. Vaccinations, by reducing the number of individuals susceptible to infections, represent a real “collective intervention”, causing not only a direct benefit to the single individual who is vaccinated, thanks to the acquired total or partial immunization, but also an indirect one following the creation of a safety network, in favour of unvaccinated people, which reduces the risk of contagion [4]. Furthermore, these benefits are achieved with the use of resources that appear to be much more contained than those that the VPDs have in terms of both direct (related to health care and pharmacological treatments and/or home care of the sick person) and indirect costs (resulting from the loss of productivity due to illness and/or disability and the human costs in terms of suffering) [5].

When Edward Jenner, back in 1797, developed the first vaccine against smallpox, he probably did not even imagine that a new and exciting era was beginning. The idea that the immunization obtained with the vaccine form of smallpox, a virus that normally caused an extremely mild form of the disease in humans, was able to make people immune to the far more serious and fearsome human form, was something completely empirical. Thanks to this vaccine, in just a century humanity has managed, for the first only time in its history, to deliberately eliminate a living species from the face of the Earth and eradicate the corresponding infectious disease [6]. The last case of human smallpox occurred in Merca, Somalia, on 26 October 1977 in a 23-year-old boy, Ali Maow Maalin. On 8 May 1980, the World Health Organization (WHO) declared at the 33rd World Health Assembly that “all the peoples of the world had been completely freed from smallpox” [7].

Modern vaccinology is a complex and multidisciplinary science which fully includes the modern knowledge of Microbiology, Immunology, Molecular Biology, Epidemiology, and Public Health. Since the 1960s, the refinement of cell culture techniques has made it possible to obtain a series of antiviral vaccines such as those against measles, mumps, and rubella [8,9]. In the 1970s and 1980s, the development of molecular biology and genetic engineering techniques made it possible to obtain increasingly effective vaccines, made up of extremely purified antigens and capable of stimulating the immune system in an ever more specific way. With these techniques, it has been possible to obtain many new vaccines, such as those against encapsulated bacteria (groups C and ACYW-135 *Neisseria* meningitidis, Streptococcus pneumoniae, and Haemophylus infuenzae), the acellular pertussis vaccine and that against hepatitis B virus (HBV), and the modern influenza subunit vaccines. The latest frontiers in the development of vaccines are reverse vaccinology and nanovaccinology, innovative techniques thanks to which some “smart” vaccines have been developed. Reverse vaccinology was specifically developed by the Italian researchers Rino Rappuoli and Maria Grazia Pizza in the Novartis laboratories in Siena [10]. This technique consists of determining the entire genomic sequence of a microorganism by genome sequencing and, then, to identify those molecules capable of functioning as potential antigens. It is an “inverse” technique since it starts not from the antigen but from the microbial genome to finally arrive at the vaccine constituent [11]. Thanks to it, it was possible to obtain the new vaccine against group B *N. meningitidis*, a remarkable success considering the epidemiological importance of this pathogen worldwide and the huge difficulties encountered, up to then, in developing an effective vaccine against it [12]. Nanovaccinology is an innovative technique using nanoparticles and nanomaterials as antigens and/or carriers of antigens, with a big capacity of stimulating immunity against them [13]. Some vaccines are now based on nanoparticles such as those against HBV and human papillomavirus (HPV). A separate mention has to be made for the very last frontier of mRNA vaccines which have been studied and used so much in the development of anti-COVID-19 vaccines and in the fight against the COVID-19 pandemic [14]. In parallel with studies on new, safer, and more effective antigens, many studies about adjuvants have been made in order to find new molecules able to effectively help antigens in strongly stimulating the immune system [15].

The COVID-19 pandemic has undoubtedly exerted a huge impact on public opinion about vaccinations for better or for worse. For sure, it’s thanks to vaccines that humanity has been able to fight against this monster and manage as best as possible a critical, unprecedented situation. However, if, on one hand, the pandemic has given a big incentive toward vaccination research, on the other hand, it has caused a recrudescence of a well-known phenomenon known as vaccine hesitancy. Paradoxically, it is the success of vaccinations that creates problems in their acceptance. It has been correctly said that “vaccinations are victims of their own success” because the decrease in the frequency of the VPDs has led to a decrease in the perception of their seriousness. In fact, over the last 30 years, there has been a progressive phenomenon of disaffection with the practice of vaccination, especially in industrialized countries. This is an expression of the progressive upsurge of anti-vaccine movements secondary, on the one hand, to the increase in the accessibility of information by the general population and, on the other hand, to the crisis of credibility and authority of healthcare professionals [16]. As one of the main points of vaccine hesitancy is the fear of side effects, the research on modern and safe antigens and adjuvants is able to play a leading role in the fight against this public health concern.

In line with all the above, this Special Issue entitled “Present and Future Vaccinations: Current Strategies and New Perspectives in the Fight Against Infectious Diseases” collects significant scientific evidence about vital vaccination topics with special regard towards some new aspects concerning the study of new antigens and adjuvants that might be able, in the future, to represent a turning point in the long and amazing history of vaccinology.
